# Two Very Interesting Cases

**Published:** 1816-05

**Authors:** Samuel Fiske

**Affiliations:** Saffron Walden, Essex.


*For the London Medical and Physical Journal.*

*Two very interesting Cases;* by SAMUEL FISKE, M.D. *of Saffron Walden, Essex.*

CASE I.—Tuesday, October 24, 1815.—John Simmons, a labouring man in this neighbourhood, aged 40, applied to me for relief for a cold and hoarseness: he breathed and swallowed with difficulty, coughed but little, and was very hoarse. He suspected, that, about a month before, he had swallowed part of the neck-bone of a fowl, and had felt a pricking and uneasiness in his throat from that time. On examining the fauces I could perceive no appearance of bone, or any extraneous substance or laceration; the tonsils and uvula were in a natural state; and, as far as I could see down the pharynx, there was no visible inflammation: externally the neck and throat, particularly the upper and right side, appeared larger and harder than natural, but gave no pain on pressure, nor could any fluid be discovered by the feel. From the peculiar noise, and difficulty in breathing and swallowing, I suspected inflammation of the larynx, and an effusion of coagulable lymph in the trachea and bronchia, or a formation of matter somewhere in the neighbouring parts. The symptoms bearing a strong analogy to the croup, a disorder which has lately proved fatal to many children here, a blister was applied to the throat, and a medicine given for his cough. The next day his breathing was much more difficult, and he could scarcely swallow; he was bled in the arm; a purge was ordered; and he was desired to inhale the steam of warm water. Thursday, a large blister was applied to the chest, and expectorating medicines given; he continued getting worse; said he felt the bone more in his throat; and died on Friday morning in great agony.

On examining the throat after death, a deep seated abscess was discovered on the upper and right side, between the larynx and pharynx, containing about four ounces of fetid pus, which, by its pressure, had nearly closed the larynx; the glottis and epiglottis were very much thickened and enlarged with an effusion of lymph; the inner membrane of the trachea was perfectly healthy, without any appearance of inflammation; the pharynx was very much thickened; and about an inch down the œsophagus was a stricture that would scarcely admit a goose quill; there was no appearance of inflammation or injury of the internal surface of the œsophagus, or any extraneous substance or spicula of bone to be discovered: the muscles of the throat covering the abscess were much thickened, and had a cartilaginous appearance.

Case II.—November 27th, 1815.—Sarah Cornel, 22 years age, near four months pregnant of her first child; a strong hearty woman, but heavy countenance, was attacked with erysipelas in the left arm, with slight fever, sickness, and costiveness; some brisk purges were given, and she was ordered to be kept warm: after three days a free evacuation from the bowels was procured; the erysipelas extended itself over the body and lower limbs; she gradually got better; and, at the end of seven days, was quite well. January 6, 1816, she complained of head-ache and dimness, with slight sickness, which continued all the 7th, (was uncertain whether she had quickened, but was supposed to be about five months advanced in her pregnancy); the 8th, in the morning, the sickness and dimness were much worse; and, at three in the afternoon, she was attacked with vomiting, blindness, and slight convulsions; she was put to bed, bled in the arm, and had hot flannels applied to the feet: it was with much difficulty she swallowed any thing. In the evening the convulsions were stronger, and she was insensible: an anodyne dose was given. At three in the morning of the 9th she was worse: another anodyne was given. On laying the hand on the abdomen, no motion of the child could be perceived, but a very strong pulsation; she had no labour pains, nor signs of labour. An examination *per vaginam* was passed: the os uteri was in its natural situation, firmly closed, and the neck of the uterus not stretched;—no attempt, therefore, was made to deliver her. She could swallow nothing, and was constantly convulsed. At nine she was evidently dying, and continued in strong convulsions till eight in the evening, when she died undelivered.

*March* 8, 1816.

